# Spontaneous knot formation between nasogastric tube and temperature probe in the oesophagus

**DOI:** 10.1002/anr3.70030

**Published:** 2025-09-24

**Authors:** D. Lindsay, M. Welstand‐Patel, N. Kalra

**Affiliations:** ^1^ Division of Anaesthesia, School of Clinical Medicine University of Cambridge Cambridge UK; ^2^ Department of Clinical Neurosciences University of Cambridge Cambridge UK; ^3^ Department of Anaesthesia Cambridge University Hospitals NHS Foundation Trust Cambridge UK

**Keywords:** equipment failure, gastrointestinal intubation, thermometers

## Report

We report a case of spontaneous intra‐operative granny knot formation between a large bore nasogastric tube (NGT) and an oro‐oesophageal temperature probe within the oesophagus [1]. Whilst entanglement between NGT and other devices has been reported; true knot formation is rare.

A 67 year‐old man presented with a sigmoid volvulus and abdominal compartment syndrome and was listed for exploratory laparotomy. After previous failed attempts in the emergency department, a 16 Fr NGT was inserted by ICU staff. However, it was observed that the NGT was not draining anything passively or on suctioning. Upon attempting to replace the NGT, resistance was encountered along with associated movement of the temperature probe.

**Figure 1:**
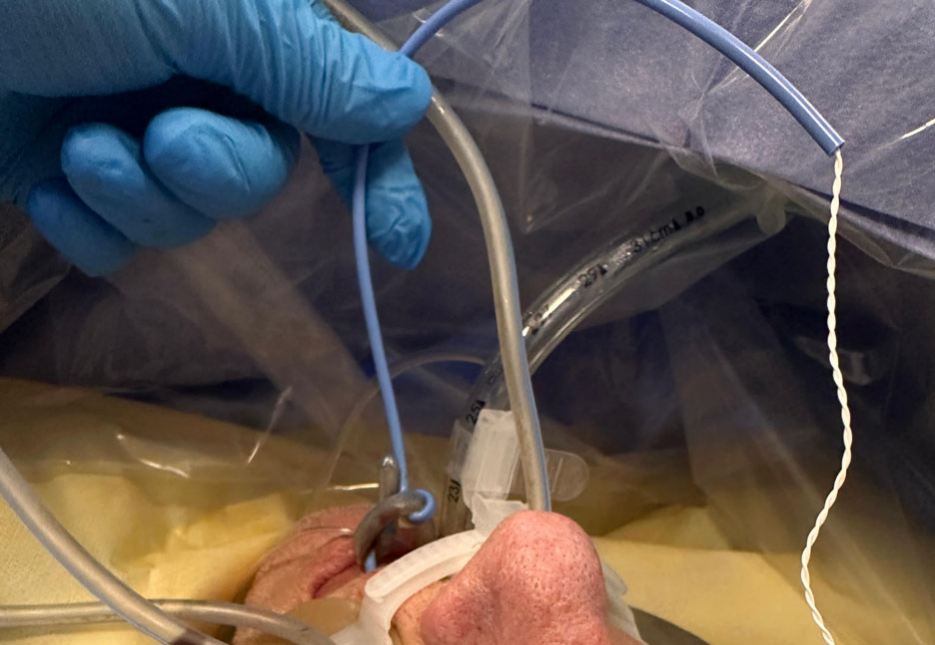
Granny knot between nasogastric tube and temperature probe.

**Figure 2:**
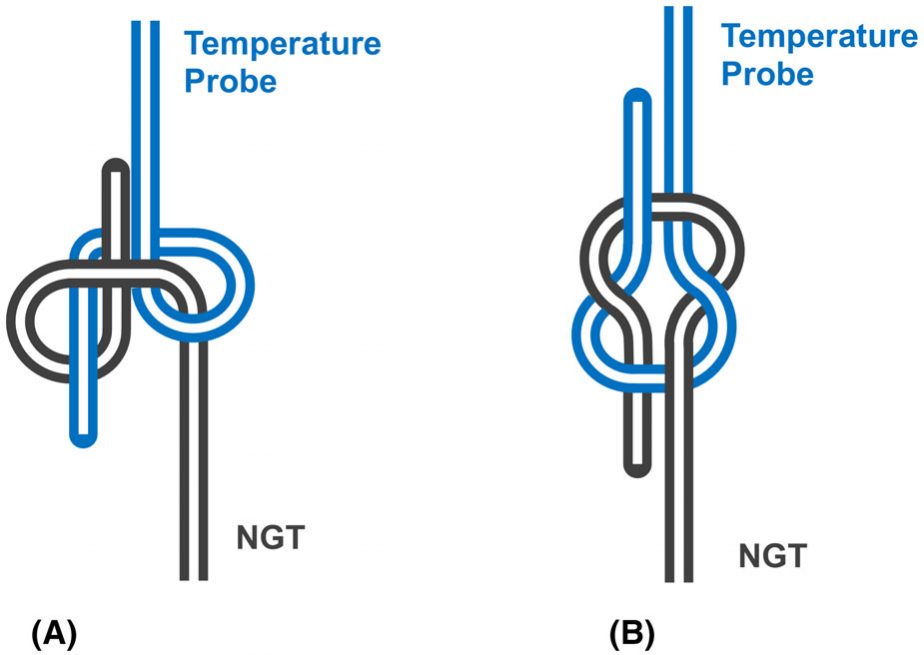
Schematic diagram of knot: (A) morphology as extracted and (B) untwisted. NGT, nasogastric tube.

Re‐advancement of the NGT and withdrawal of the temperature probe allowed the knot to be visualised and undone (Figures 1 and 2). While cutting the NGT and extracting the knot en bloc would have been an option, releasing the knot and extracting the devices intact reduced the risk of soft tissue trauma from the cut end.

The granny knot is related to the surgeon's knot and reef knot commonly used in surgical suturing techniques. It can be used as a binding knot, such as in a simple suture encircling opposed edges of a wound, or as a bend to join the ends of two lengths. However, the latter is insecure as it may capsize under tension unless externally stabilised, leading it to become undone [2]. In this instance, we believe it may have been stabilised in a bend configuration by the surrounding tissues of the oesophagus.

## References

[1] Davey AJ , Diba A . Ward's Anaesthetic Equipment. 6th ed. Saunders, Philadelphia, PA: 2012.

[2] Patil VP , Sandt JD , Kolle M , Dunkel J . Topological mechanics of knots and tangles. Science 2020; 367: 71–75. 31896713 10.1126/science.aaz0135

